# Crystal structure of an HD‐GYP domain cyclic‐di‐GMP phosphodiesterase reveals an enzyme with a novel trinuclear catalytic iron centre

**DOI:** 10.1111/mmi.12447

**Published:** 2013-11-24

**Authors:** Dom Bellini, Delphine L. Caly, Yvonne McCarthy, Mario Bumann, Shi‐Qi An, J. Maxwell Dow, Robert P. Ryan, Martin A. Walsh

**Affiliations:** ^1^ Diamond Light SourceHarwell Science and Innovation CampusDidcotOxfordshireOX11 0DEUK; ^2^ Research Complex at HarwellHarwell Science and Innovation CampusDidcotOxfordshireOX11 0FAUK; ^3^ Department of MicrobiologyBiosciences InstituteUniversity College CorkCorkIreland; ^4^ Department of Chemistry and BiochemistryUniversity of BernCH‐3012BernSwitzerland; ^5^ Division of Molecular MicrobiologyCollege of Life SciencesUniversity of DundeeDow streetDundeeDD1 5EHUK

## Abstract

Bis‐(3′,5′) cyclic di‐guanylate (c‐di‐GMP) is a key bacterial second messenger that is implicated in the regulation of many crucial processes that include biofilm formation, motility and virulence. Cellular levels of c‐di‐GMP are controlled through synthesis by GGDEF domain diguanylate cyclases and degradation by two classes of phosphodiesterase with EAL or HD‐GYP domains. Here, we have determined the structure of an enzymatically active HD‐GYP domain protein from *Persephonella marina* (*Pm*GH) alone, in complex with substrate (c‐di‐GMP) and final reaction product (GMP). The structures reveal a novel trinuclear iron binding site, which is implicated in catalysis and identify residues involved in recognition of c‐di‐GMP. This structure completes the picture of all domains involved in c‐di‐GMP metabolism and reveals that the HD‐GYP family splits into two distinct subgroups containing bi‐ and trinuclear metal centres.

## Introduction

Bis‐(3′,5′) cyclic di‐guanylate (c‐di‐GMP) is a second messenger utilized by almost all eubacteria that acts to regulate a wide range of functions including developmental transitions, adhesion, biofilm formation, motility and the synthesis of virulence factors (Schirmer and Jenal, 2009; Boyd and O'Toole, 2012). C‐di‐GMP is synthesized from two GTP molecules by GGDEF domain‐containing diguanylate cyclases (DGCs) and degraded by phosphodiesterases (PDEs) with either an EAL or HD‐GYP domain (Ryan *et al*., 2006; Hengge, 2009; Schirmer and Jenal, 2009; Boyd and O'Toole, 2012). Three‐dimensional structures have been determined for GGDEF and EAL domains, and have afforded detailed insight into their roles in the turnover of c‐di‐GMP and regulatory interactions with other proteins (Hengge, 2009; Schirmer and Jenal, 2009; Boyd and O'Toole, 2012). In contrast, enzymatically active HD‐GYP domain proteins, such as the paradigm RpfG from the plant pathogen *Xanthomonas campestris* (Ryan *et al*., 2006), have so far proved intractable to structure determination by X‐ray diffraction.

The structure of an unconventional catalytically inactive HD‐GYP domain protein from *Bdellovibrio bacteriovorans* (Bd1817) has been determined however (Lovering *et al*., 2011). This work identified a binuclear iron centre and the role of conserved residues within the HD‐GYP family (to include the HD diad) in metal binding. The HD domain superfamily of enzymes, to which the HD‐GYP family belongs, has been shown to catalyse phosphomonoesterase and phosphodiesterase reactions depending on their catalytic metal centre being mono‐ or binuclear respectively (Aravind and Koonin, 1998; Galperin *et al*., 2001). The determination of the structure of Bd1817 may thus afford some insight into metal binding by enzymatically active HD‐GYP domains, but the protein lacks the conserved tyrosine of the GYP motif and has no c‐di‐GMP phosphodiesterase activity, precluding insights into the role of the other conserved residues.

Here we describe the first crystal structure of an enzymatically active HD‐GYP phosphodiesterase protein, *Pm*GH from *Persephonella marina* EX‐H1, a thermophilic marine member of the Aquificales. *Pm*GH comprises an HD‐GYP domain fused to a GAF domain. We have also determined structures for this protein in complex with its substrate (c‐di‐GMP) and final reaction product (GMP). The structures reveal the mode of binding of the di‐nucleotide and shed light on the catalytic mechanism. A remarkable feature of the structure of *Pm*GH was the identification of a trinuclear Fe centre which is buried at the bottom of the cavity forming the c‐di‐GMP binding site. Adequate space is available for the substrate to bind dynamically and interact with the metal centre to sequentially hydrolyse c‐di‐GMP to GMP. Knowledge of the conserved residues involved in binding to the novel trinuclear Fe centre together with the analysis of amino acid sequence alignment of a large cohort of HD‐GYP domain proteins suggests further classification of this family into two distinct subgroups containing either a bi‐ or trinuclear metal centre.

## Results

### Target selection and structure determination

**Figure 1:**
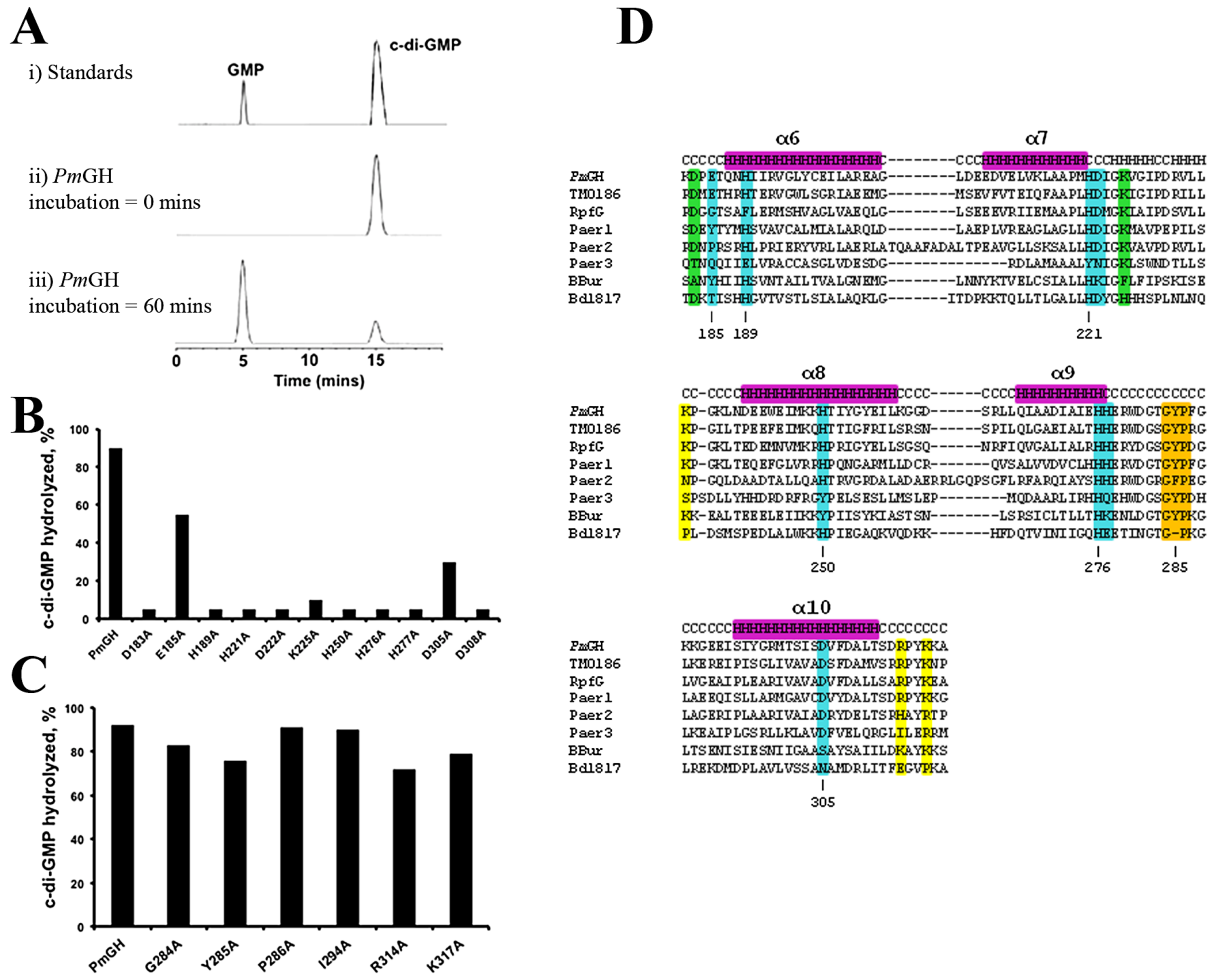
PDE activity of purified HD‐GYP domain *Pm*GH and variants with alanine substitutions. A. Representative HPLC traces showing standards (i), aliquots of reaction mixtures boiled at 0 min (ii) and after 60 min (iii) incubation with the *Pm*GH protein. The identity of the product was confirmed by mass spectrometry. B. Effects of alanine substitutions in metal ligands (E185, H189, H221, D222, H250, H276, H277 and D305), a strongly conserved residue in the family (D308) and the two putative catalytic residues (D183 and K225) on cyclic di‐GMP hydrolysis. C. Effects of alanine substitutions in residues in the GYP motif (G284, Y285 and P286), the conserved I294 position and other residues involved in substrate binding (R314 and K317) on cyclic di‐GMP hydrolysis. D. Primary sequence alignment of HD‐GYP domains of *Pm*GH with some of the most well‐characterized HD‐GYP proteins, such as TM0186 (*Thermotoga maritima*), RpfG (*Xanthomonas campestris* pv. *campestris*), Paer1‐3 (*Pseudomanas aeruginosa*), BBur (*Borrelia burgdorferi*) and Bd1817 (*Bdellovibrio bacteriovorus*). Metal ligands, catalytic residues, substrate ligands and GYP motif, based on the *Pm*GH structure, are highlighted in cyan, green, yellow and orange respectively.

Initial attempts to crystallize the archetypal HD‐GYP domain protein RpfG from *Xanthomonas campestris* (Ryan *et al*., 2006) failed. An extensive search of structural homologues with standalone HD‐GYP domains and/or in combination with different sensor and ligand binding domains was then made; this extensive list was rationalized through bioinformatics analysis using criteria known to increase the success rate of crystallization (Slabinski *et al*., 2007). Of a cohort of 15 proteins, a GAF/HD‐GYP domain‐containing protein from *P. marina* EX‐H1 (*Pm*GH) gave crystal hits that were further optimized for structure solution. *Pm*GH was shown to have c‐di‐GMP phosphodiesterase activity both in the crystalline form (see below) and in solution (Fig. 1). The structure was solved by the single wavelength anomalous diffraction (SAD) technique using the anomalous signal from the bound iron (Table 1).

**Table 1:** Data collection and refinement statistics

	*Pm*GH Fe peak	*Pm*GH native	*Pm*GH c‐di‐GMP	*Pm*GH GMP
Data collection				
X‐ray source	Diamond I24	Diamond I02	Diamond I02	Diamond I04‐1
Wavelength (Å)	1.74	0.95	0.98	0.92
Resolution range (Å)^a^	71–2.70 (2.8–2.70)	72–2.03 (2.1–2.03)	66–2.68 (2.75–2.68)	71–2.55 (2.62–2.55)
Space group	I222	I222	I222	I222
Unit cell parameters				
a, b, c (Å)	69.7, 180.8, 232.2	69.8, 182.4, 232.5	70.2, 183.0, 233.5	70.1, 181.1, 231.5
α, β, γ (°)	90, 90, 90	90, 90, 90	90, 90, 90	90, 90, 90
No. of observations	102 579	417 588	189 599	254 040
No. of unique observations	38 405	95 384	42 290	48 066
* R*_merge_ (%)	12.2 (72.1)	4.1 (56.0)	7.7 (68)	8.6 (68)
Completeness (%)^a^	98.7 (97.3)	99.8 (98.0)	99.4 (99.6)	99.2 (98.7)
Mean *I/σI*^a^	28.3 (5.3)	15.2 (2.3)	14.3 (1.9)	12.7 (2.2)
B wilson	–	52.42	69.14	60.06
Refinement				
* R*_work_/*R*_free_ (%)	–	19/21	18/23	18/22
Rms deviations, bonds/angles	–	0.011/1.36°	0.013/1.72°	0.010/1.42°
Average B factor (Å^2^)	–	52.8	45.2	37.3
Ramachandran favoured	–	96.8%	96.1%	96.4%

a Values in parentheses refer to the high resolution shell. Anomalous pairs were kept separate during merging of all datasets.

### Protein architecture

**Figure 2:**
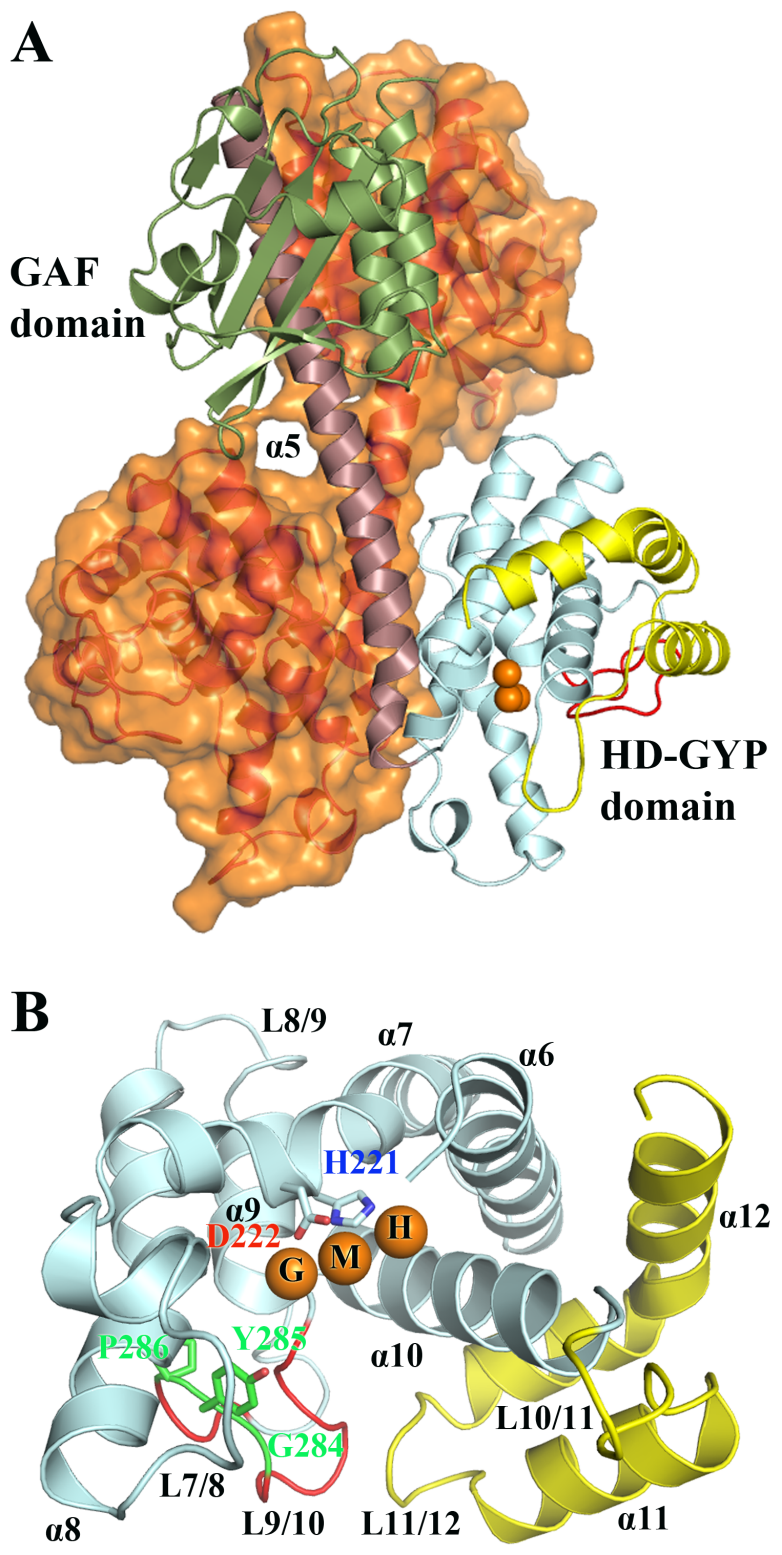
Structure of *Pm*GH. A. Structure of the *Pm*GH homodimer. Molecule A of the dimer is shown in ribbon representation with the GAF domain in green, the long inter‐domain dimerization helix α5 in maroon, the core HD domain in cyan, the GYP motif‐containing loop in red and the additional surface decorating α‐ helices which complete the HD‐GYP domain in yellow. Molecule B of the dimer is shown in ribbon representation in orange with a semi‐transparent surface. The trinuclear iron centre is shown as orange spheres. B. Detailed view of the HD‐GYP domain of *Pm*GH in ribbon presentation. Colour codes as in (A) with the addition of the HD and GYP motif residues shown in ball and stick. Labelling for α‐helices and turns are shown. The central metal iron has been labelled as the middle site (M) and the two flanking metal sites as H and G, to reflect their proximity to the HD and GYP motifs respectively.

*Pm*GH forms a dimer with each monomer consisting of an N‐terminal GAF domain connected to a C‐terminal HD‐GYP domain by an approximately 42 residue‐long helix (α5 in Fig. 2A). Assembly of the head‐to‐head dimer relies exclusively on the GAF domain and the long α5 helix with the HD‐GYP domain playing no role in the dimeric interface (Fig. 2A). The overall topology of the GAF domain is similar to that of other GAF domain structures (Ho *et al*., 2000; Kanacher *et al*., 2002; Martinez *et al*., 2002), consisting of a six‐stranded antiparallel β‐sheet (β3‐β2‐β1‐β6‐β5‐β4) sandwiched by a three‐helix bundle (α1, α2 and α5) on one side and two short helices (α3 and α4) on the other.

**Figure 3:**
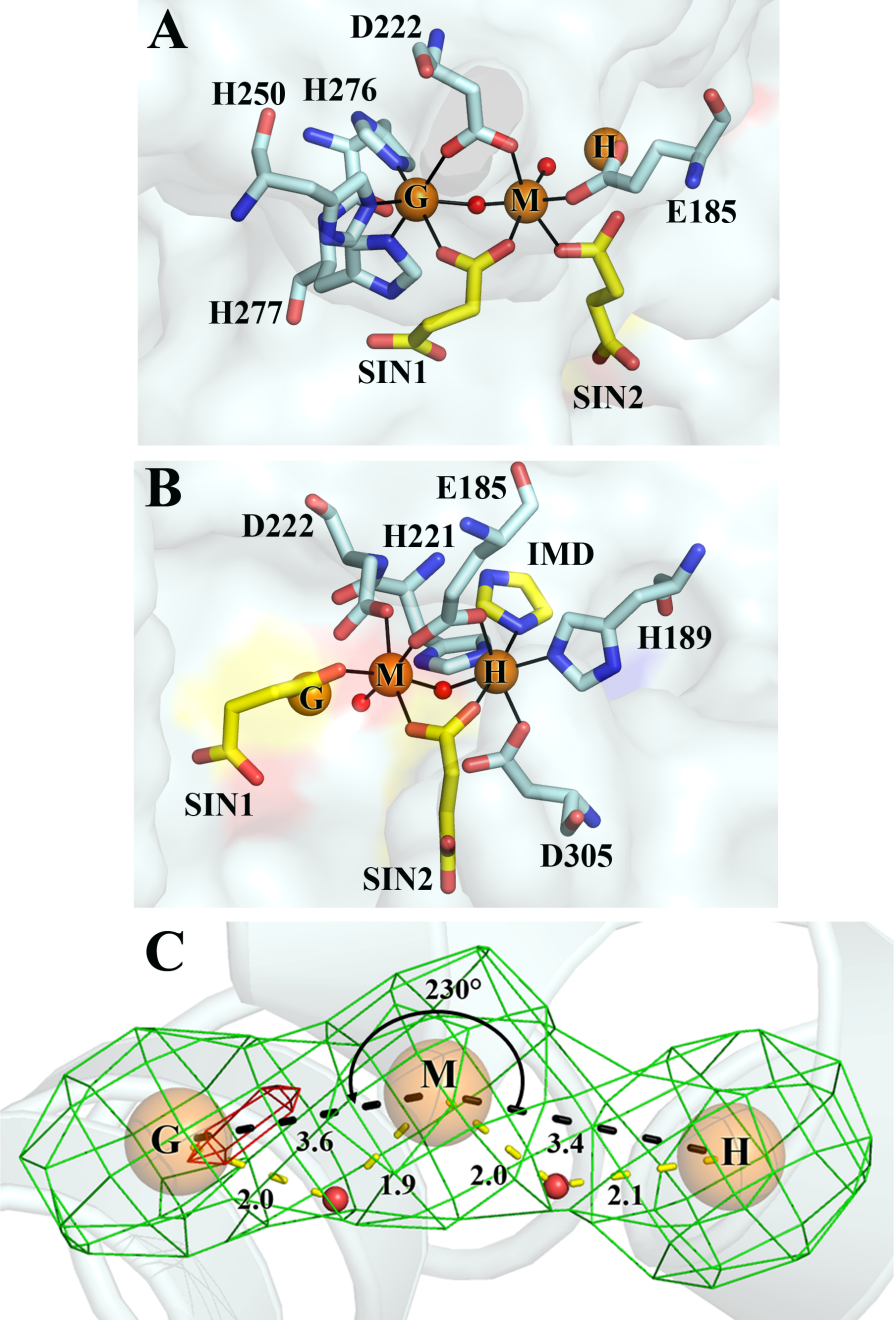
The tri‐iron metal centre of *Pm*GH. Detailed views of the tri‐iron centre showing the first co‐ordination sphere for the G‐M metal pair (A) and for the M‐H metal pair (B) with the co‐ordination of the H and G sites not shown in (A) and (B) respectively for clarity. Protein metal interactions are highlighted as black lines. Fe atoms are shown as orange spheres. Protein side‐chain metal ligands are in stick mode, coloured by atom type with carbon in pelican, while the carbon atoms of the metal ligands from the crystallization buffer, 2 succinates SIN‐1,2 and an imidazole ion (IMD), are in yellow. (C) Fe‐specific difference DANO map (Than *et al*., 2005) in green and Mn anomalous difference map in red, both contoured at 0.043 eÅ‐3. The angle subtended by the tri‐iron centre is shown. Black dashed lines depict Fe–Fe distances, while yellow dashed lines indicate bond distances for the pair of μ‐hydroxides. Bond distances are in Ångstroms.

The catalytic HD‐GYP domain contains the characteristic HD domain superfamily 5‐helix core formed by helices α6 to α10 that in turn provides the scaffold for sequestering the tri‐iron centre through eight conserved protein side‐chain ligands (Fig. 2B and Fig. 3). The signature HD motif forms part of this octet (H221 and D222) and is located on α7 at a kink close to the C‐terminal end of the helix. Another four conserved histidines provide metal ligands through H189 located at the start of α6, H250 from α8 and H276/277 at the end of α9. D305 from α10 completes the protein metal ligands of the tri‐iron centre. In addition to the HD domain 5‐helix core, the HD‐GYP domain contains two extra C‐terminal helices, a short helix α11 and finally α12 which is significantly bent and allows it to pack against helices α6 and α10 (Fig. 2B).

The HD‐GYP domain of *Pm*GH in its entirety resembles an opened two‐clawed chela. One of the chela's claws is comprised of the loop (L7/8) connecting α7 and α8 as well as the start of α8, whereas the other claw is entirely formed by the loop connecting helices α10 and α11 (L10/11) (Fig. 2B). The loop region connecting helices α9 and α10 contains the signature GYP motif and forms a well‐ordered structure made up of two orthogonally orientated U‐turns as observed in the structure of an inactive HD‐GYP domain protein (Lovering *et al*., 2011). The conserved Y285 of the GYP motif points towards the metal binding centre which is buried in the cavity formed by the chela. The ‘GYP’ loop forms a barrier on one side of the opened chela, whereas the other side is unobstructed. A sequence‐based conserved motif in HD‐GYP proteins mapping to loop L9/10 was previously identified to be HHExxDGxGYP (Ryan *et al*., 2006); however, the *Pm*GH structure reveals that L9/10 is composed of 19 residues suggesting an extension of the consensus sequence to HHExxDGxGYPxxxxxxxI, which includes a conserved isoleucine residue (I294 in *Pm*GH) that stabilizes the closure of the second U‐turn by hydrophobic interactions with G284 from the GYP motif.

### Trinuclear Fe binding site

The HD‐GYP domain of *Pm*GH contains a trinuclear metal centre that is located at the bottom of the cavity formed by the two claws of the open chela (Fig. 2B; Fig. 3A and B). Anomalous diffraction difference maps showed all three metal sites to be occupied by iron atoms, with anomalous peak heights in excess of 40 σ separated by approximately 3.5 Å (Fig. 3C). The bond valence sum method (Brown and Altermatt, 1985) was used to estimate the oxidation states of the three Fe ions which assigned the two peripheral sites as occupied by Fe(II) and the middle site by Fe(III). Overexpression of protein from bacteria grown in minimal media and supplementing with divalent metal ions such as manganese resulted in mixed metal occupancies in the peripheral metal sites but the middle metal site was always observed to be occupied by Fe (Fig. 3C and Fig. S3). These data support the oxidation state assignment and indicate that the middle Fe site of the trinuclear centre is specific for Fe(III), while the peripheral sites can accommodate other metals under Fe deficient conditions. The tri‐iron centre delineates the floor of the open chela with the two peripheral metal sites being in close proximity to Y285 of the GYP motif on one side (the G‐site) and H221 of the HD motif on the other side (the H‐site). The middle iron binding site (M‐site) is sandwiched between the G‐ and H‐sites with Fe‐Fe bond distances of 3.40 Å and 3.67 Å respectively (Fig. 2B and Fig. 3). All three metals are octahedrally co‐ordinated (Fig. 3A and B). In *Pm*GH the HD‐GYP domain conserved residues E185, H189, H221, D222, H250, H276, H277 and D305 contribute to ligand binding with the metal co‐ordination sphere completed by succinate and imidazole bound from the crystallization buffer as well as two solvent molecules which bridge the G‐M and M‐H metal pairs (Fig. 3 and Fig. S1). Both pairs of Fe sites, G‐M and M‐H, are triply bridged: the G‐M pair by the carboxylate groups of D222 and a succinate from the crystallization buffer in a bidentate fashion and by a monodentate bridged solvent molecule; whereas the M‐H pair is bidentately co‐ordinated by the carboxylate groups of E185 and a second succinate ion and by another monodentate bridged solvent molecule (Fig. 3A and B). Analysis of metal‐ligand bond lengths for the bridging solvent molecules is consistent with the bridging ligand being a hydroxide ion. This correlates with other HD domains containing diiron centres (Brown *et al*., 2006; Lovering *et al*., 2011).

### C‐di‐GMP and GMP binding

**Figure 4:**
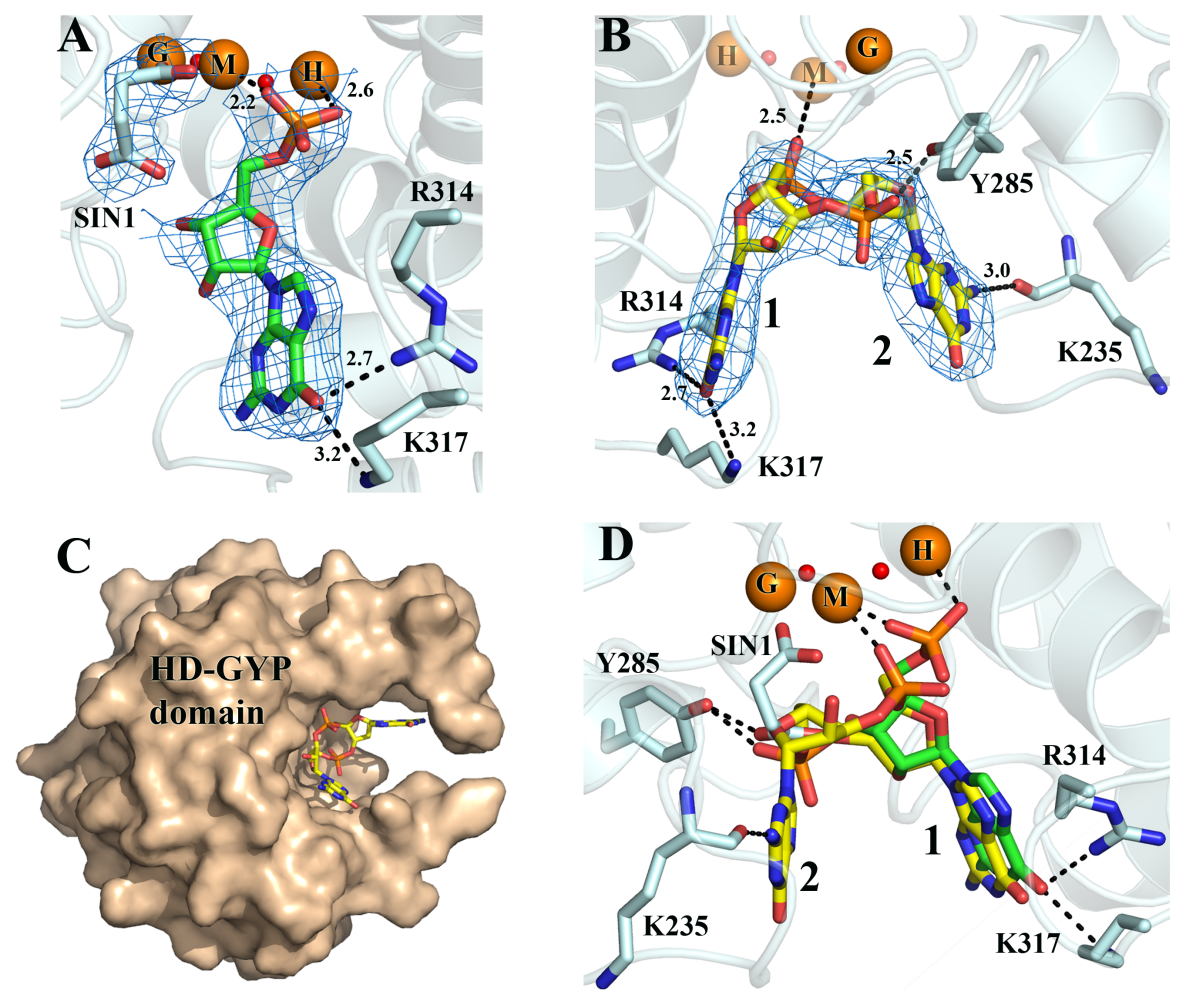
Substrate binding by *Pm*GH. A. View of GMP shown in stick mode and coloured by atom type bound to *Pm*GH. Bonding interactions are represented by dashed lines with distances in Angstroms. Difference electron density map for GMP is contoured at 2 σ. B. View of cyclic di‐GMP bound to a metal depleted subunit of *Pm*GH. Electron density for cyclic di‐GMP is from a bias‐removed omit map contoured at 2 σ. Fe atoms occupying the middle (M) and HD (H) sites are shown as semi transparent spheres as they are not present in the subunit (due to chelation by EDTA), and are taken from superposition of the equivalent metals from the high resolution structure of *Pm*GH. C. Surface representation of the *Pm*GH HDGYP domain monomer subunit showing the binding cavity for cyclic‐di‐GMP, which is represented in stick mode and coloured by atom type. D. Superposition of the structures of *Pm*GH bound to cyclic di‐GMP and GMP. Both nucleotides are shown in stick mode.

Structures of complexes of *Pm*GH with GMP and c‐di‐GMP were determined from crystal soaking experiments with both nucleotides. Crystal soaks with GMP and c‐di‐GMP gave identical structures of *Pm*GH in complex with GMP showing that *Pm*GH retained phosphodiesterase activity in the crystal (Fig. 4A). However, pre‐soaking of *Pm*GH protein crystals in the presence of 100 mM EDTA followed by c‐di‐GMP revealed well defined difference electron density for a bound c‐di‐GMP molecule at the active site, displacing the metal bound succinate ions found in the nucleotide free structure. The c‐di‐GMP was modelled unambiguously with the two guanines in a *cis* conformation so that the molecule presents a V‐shaped conformation when bound to *Pm*GH (Fig. 4B). This is in contrast to what is observed in EAL domain proteins where c‐di‐GMP is bound in a more extended conformation (Navarro *et al*., 2009). Crystal packing presents a less solvent exposed metal binding site for one of the monomers of the *Pm*GH dimer and binding of nucleotide is observed only to monomer B. The structure reveals that only the G‐site Fe remains bound in this monomer of *Pm*GH in these EDTA treated crystals, whereas the other less accessible monomer subunit still has Fe bound at all three metal sites, but with reduced occupancy (∼ 50%) for the M and H sites and shows no difference density for bound nucleotide.

The bound c‐di‐GMP is buried within the large pocket formed primarily by the HD‐GYP domain claws (65% of its accessible surface, 523 Å^2^ buried), with one of the phosphate groups pointing towards the tri‐iron centre, while the ribose and guanine bases are stacked against the claws of the chela (Fig. 4B and C). Superposition of the *Pm*GH monomer bound to c‐di‐GMP, which has only the G‐site Fe occupied, onto the tri‐iron *Pm*GH structure shows the *Pm*GH c‐di‐GMP complex structure to be essentially unchanged on binding of c‐di‐GMP apart from E185 which is disordered due to the loss of the M‐H Fe metal pair as well as better defined density for the residues of loop L10/11 which interact directly with the bound c‐di‐GMP. This analysis shows the bound c‐di‐GMP to interact with the middle Fe(III) (M‐site) through one of the non‐bridging phosphate oxygens of one hydrolysable phosphate group and to the hydroxyl group of Y285, the signature Y residue of the GYP motif, through a hydrogen bonding interaction with a non‐bridging oxygen of the other hydrolysable phosphate (Fig. 4B). One of the guanine bases, base‐1, forms three hydrogen bonds to *Pm*GH, two through the C6 carbonyl oxygen of the guanine with the NH1 guanidinium group of R314 and the NZ amine nitrogen of K317 and a third through the guanine N7 atom and the NH1 guanidinium group of Arg‐314 (Fig. 4B). Guanine base‐2 on the other hand interacts with *Pm*GH only through the C2 amine group that makes a hydrogen bond to the main‐chain carbonyl of K235. Hydrophobic interactions with Y44, A309 and L310 complete the binding interactions of *Pm*GH with c‐di‐GMP (Fig. S2). Comparison of GMP and c‐di‐GMP binding shows that the guanine base of the GMP molecule superposes with the guanine base‐1 of c‐di‐GMP (Fig. 4D). In the case of the tri‐iron *Pm*GH‐GMP complex, the GMP phosphate moiety bridges the M and H metal sites as opposed to the inferred sole interaction of one of the hydrolysable phosphate groups with the middle Fe M‐site based on the crystal structure of the mononuclear Fe *Pm*GH‐c‐di‐GMP complex (Fig. 4A and D).

### Mutational analysis of the role of key residues

Alanine substitutions of metal ligands in *Pm*GH (E185A, H189A, H221A, D222A, H250A, H276A, H277A and D305A) essentially abolished the phosphodiesterase activity in all cases except for E185 and D305, where although there is a marked reduction in their ability to hydrolyse c‐di‐GMP, hydrolysis of c‐di‐GMP is still detected (Fig. 1B). Mutations in the GYP motif and conserved residues implicated in c‐di‐GMP recognition (G284A, Y285A, P286A, I294A, R314A and K317A) did not however result in a substantial decrease in catalytic activity (Fig. 1C). Alanine mutation of other conserved residues near the metal centre (D183, D308 and K225) had a similar impact on activity as for metal ligand residues. D308 is located at the C‐terminal of helix α10 forming hydrogen bonds with R192 at the N‐terminal of α5 and the metal ligand H189 highlighting a structural role in stabilizing the metal centre and the HD fold, while K225 contributes to stabilization of the tri‐iron centre through hydrogen bonds with the metal ligands E185, D222 and also D183 (Fig. 1B).

## Discussion

HD‐GYP domain‐containing proteins are a large family of the HD superfamily of metal‐dependent phosphohydrolases (Aravind and Koonin, 1998). The predicted role of the HD‐GYP domain as a PDE active against c‐di‐GMP (Galperin *et al*., 2001) was first demonstrated for RpfG from *X. campestris* (Ryan *et al*., 2006; Ryan, 2013) with further examples characterized from other bacteria such as *Pseudomonas aeruginosa* (Ryan *et al*., 2009; Stelitano *et al*., 2013), *Borrelia burgdorferi* (Sultan *et al*., 2011), *Thermotoga maritima* (Plate and Marletta, 2012) and *Vibrio cholerae* (Miner *et al*., 2013). The crystal structure of a HD‐GYP protein from *P. marina* (*Pm*GH) and its complex with c‐di‐GMP provides the first structure of an active HD‐GYP domain protein that reveals a trinuclear Fe centre. The mode of binding of the cyclic nucleotide differs significantly from that observed for the more extensively characterized EAL domain containing PDEs (Schirmer and Jenal, 2009) while the binding site provides adequate room to allow both hydrolysable phosphates to interact in turn with the metal centre to complete hydrolysis of the c‐di‐GMP to GMP. The mode of c‐di‐GMP binding to the tri‐iron site extends the diversity of structure and function of bacterial c‐di‐GMP phosphodiesterases.

### Structural and chemical relationships of the trinuclear Fe centre

**Figure 5:**
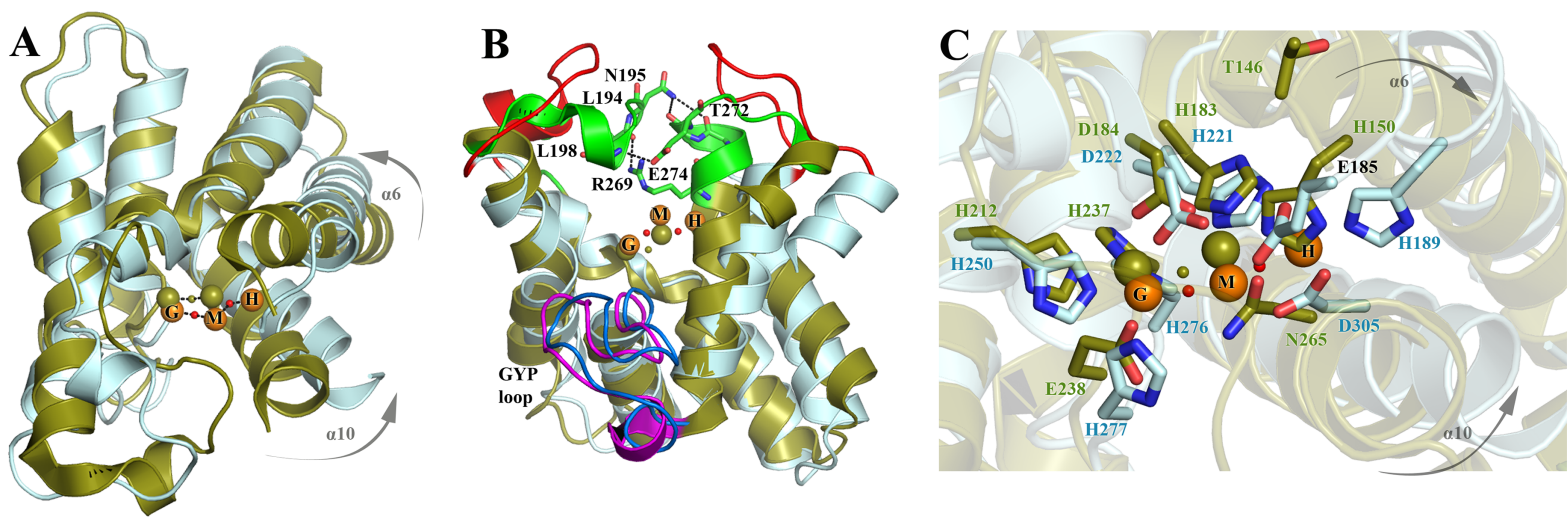
Comparison of the structures of the HD‐GYP domain from *Pm*GH and the unconventional HD‐GYP domain of Bd1817. A. *Pm*GH and Bd1817 PDE domains are shown in ribbon representation and coloured cyan and olive green respectively. The *Pm*GH metal centre is as for Fig. 1, while the Bd1817 binuclear Fe centre with a bridging hydroxide ion is shown as green spheres. The shift in orientation in helices α6 and α10 in *Pm*GH when compared with Bd1817 which allows the protein to accommodate the trinuclear metal centre is highlighted. B. Comparison of the HD‐GYP domain nucleotide‐binding pocket highlighting the opened and closed conformations observed for *Pm*GH and Bd1817 in red and green respectively. Hydrogen bonds proposed to hold the Bd1817 in this close conformation are shown as black dashed lines. The double headed black arrow depicts the distance in Angstroms between the L7/8 and L10/11 loops in *Pm*GH for which the active site is in an open conformation. C. Secondary structure superposition using protein fragments ranging from first to last residue involved in metal co‐ordination of *Pm*GH and Bd1817 showing only the protein metal ligands.

Although all HD domains share key design features, a striking diversity of catalytic centres have now been identified, containing no metal, mono‐ or binuclear metal centres, and here a trinuclear metal binding site. In *Pm*GH the tri‐iron site presents a structure which is distinct from the more extensively studied oxo‐centred equilateral triangle trinuclear iron complexes, although the first protein structure with such an oxo‐centred iron cluster was only determined relatively recently (Hogbom and Nordlund, 2004). Here, the tri‐iron centre of *Pm*GH has striking similarities to a series of linear tri‐iron complexes that were obtained while designing mimetics for studying primarily carboxylate‐bridged bimetallic centres in metalloproteins (Rardin *et al*., 1992; Kitajima *et al*., 1993). However in *Pm*GH the tri‐iron centre has an extended V shape (Fig. 3C). The G and M metal sites of the tri‐iron centre align closely with the diiron metal centre of Bd1817, an inactive HD‐GYP protein (Lovering *et al*., 2011) (Fig. 5A) and that of other HD domain containing binuclear Fe sites such as myo‐inositol oxygenase (Brown *et al*., 2006). Comparison of the structure of *Pm*GH to Bd1817 shows the overall fold of the HD‐GYP domain is maintained but reveals a slight re‐orientation of the first and fifth α‐helices of the 5‐helix HD core domain to accommodate the tri‐iron centre (Fig. 5A and C). *Pm*GH presents an accessible and far larger binding cavity than is the case for Bd1817 where the active site is capped by a ‘lid’ formed by loops corresponding to L7/8 and L10/11 in *Pm*GH (Fig. 5B). The Bd1817 interactions responsible for keeping the lid tightly fixed in place are unique to Bd1817 (Fig. 1D), suggesting that these mutations have evolved to intrinsically fix the lid in a closed conformation to prevent binding of nucleotide keeping the protein enzymatically inactive. Despite the fact that a significant number of enzymatically inactive EAL and HD‐GYP domains have been demonstrated to play a role in signal transduction and regulation through c‐di‐GMP binding and/or protein interaction (Navarro *et al*., 2009; Ryan *et al*., 2009), the regulatory role of Bd1817 still remains to be revealed (Lovering *et al*., 2011).

**Figure 6:**
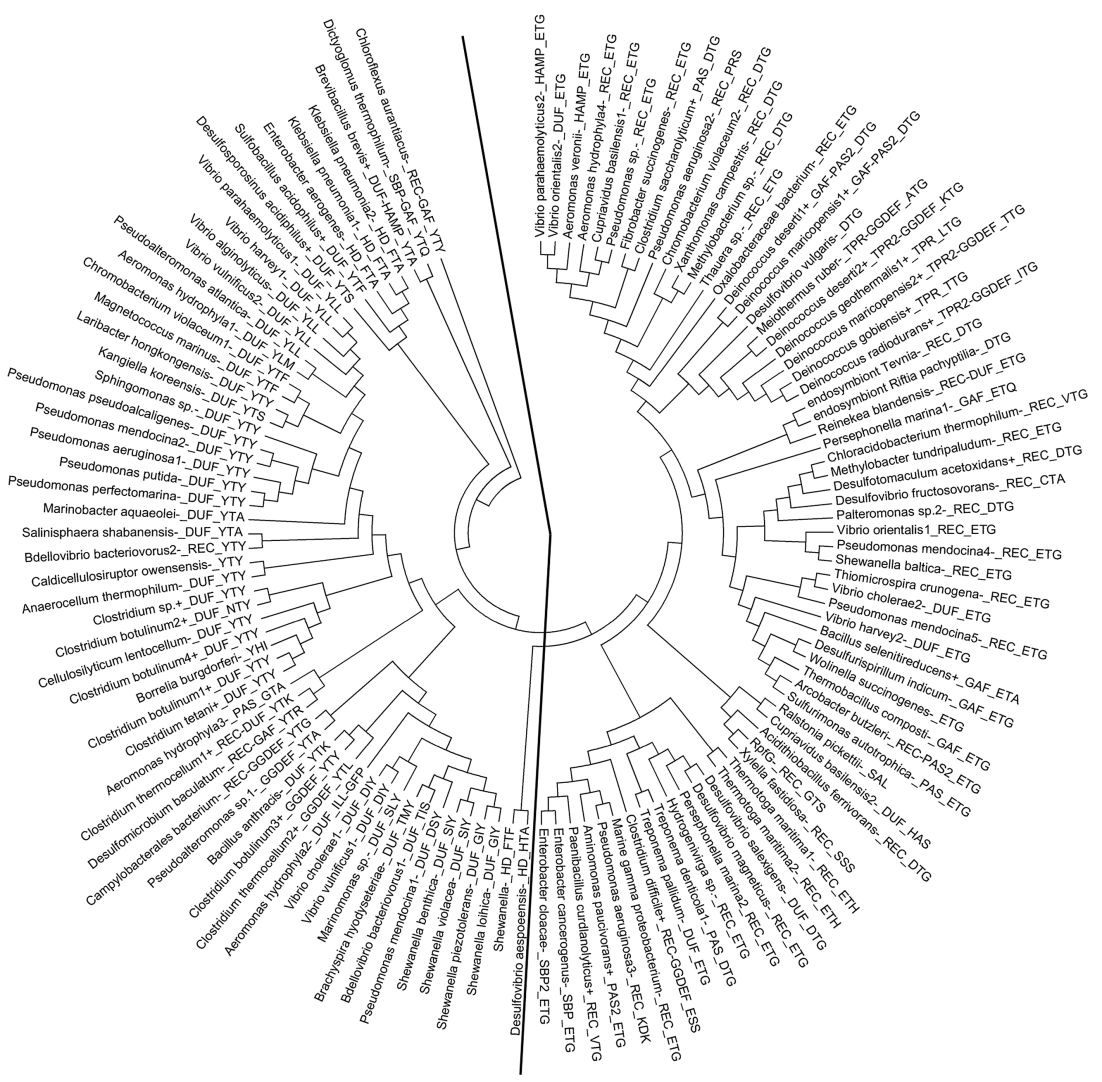
Maximum parsimony analysis of various HD‐GYP domain sequences. The evolutionary history was inferred using the maximum parsimony (MP) method. Tree #1 out of 17 most parsimonious trees (length = 3871) is shown. The consistency index is 0.257040 (0.255115), the retention index is 0.444680 (0.444680), and the composite index is 0.114300 (0.113445) for all sites and parsimony‐informative sites (in parentheses). The MP tree was obtained using the Close‐Neighbor‐Interchange algorithm (Suzuki *et al*., 2002) with search level 0 in which the initial trees were obtained with the random addition of sequences (10 replicates). The analysis involved 122 amino acid sequences. All positions containing gaps and missing data were eliminated. There were a total of 116 positions in the final dataset. Sequence labels are in the following format: (1) organism name followed by a number if more than one sequence is present from the same organism; (2) a +/− sign indicating Gram + or Gram −; (3) other domains present besides the HD‐GYP domain are indicated, followed by a number in case of multiple copies; (4) a 3 letter code for the 3 residues triplet subfamily signature corresponding to positions 185–187 in *Pm*GH. Extra domains are: REC = CheYhomologous receiver domain; GAF = present in cyclic di‐GMP phosophodiesterase, Adenyl cyclase, Fhla; PAS = present in Periodic circadian protein, Ah receptor nuclear translocator protein, Single‐minded protein; HAMP = present in Histidine kinases, Adenyl cyclases, Methyl‐accepting proteins and Phosphatases; HD = extra HD domain of unknown function missing the GYP motif; TPR = domain containing the Teratrico Peptide Repeat region; GGDEF = diguanylate cyclase containing the GGDEF motif; SBP = bacterial extracellular Solute‐ Binding Protein; DUF = Domain of Unknown Function. Evolutionary analyses were conducted in Mega5 (Tamura *et al*., 2011).

Our observation of the tri‐iron centre in the HD‐GYP domain of *Pm*GH prompted us to perform a phylogenetic comparison of this domain with all relevant proteins in the National Center for Biotechnology Information (NCBI) database. This analysis showed a distinct separation of the HD‐GYP domains into two evolutionary groups (Fig. 6). This bipartition is independent of the type of regulatory and/or sensory domain associated with the HD‐GYP domain (Fig. 6). Analysis of the sequence alignments highlighted that only seven out of the eight *Pm*GH metal ligand residues were shared between these two groups. The one variable ligand corresponds to E185 in *Pm*GH which provides a bidentate carboxylate ligand which bridges the M and H metal sites. In the group containing all eight ligands, which includes *Pm*GH and TM0186 from *Thermotoga maritima*, a REC/HD‐GYP protein that has been characterized as an active c‐di‐GMP phosphodiesterase(Plate and Marletta, 2012), the equivalent of the metal ligand E185 is invariably found to be conserved or replaced by an aspartate. A threonine and a glycine consistently follow this conserved metal carboxylate ligand (E/D), giving the signature motif E/D‐T‐G for this subfamily. Conversely the other subfamily primarily presents a tyrosine or phenyalanine (Y/F) in place of the conserved carboxylate ligand of E185. Despite the lack of a unique signature triplet as in the E/D‐T‐G group (except in some rare cases), there do exist some well‐populated sequence clusters within this second Y/F subfamily identifiable by motifs such as Y‐T‐Y, Y/I‐L‐L or F‐T‐F. The Y/F mutation of the carboxylate metal ligand that in the *Pm*GH structure bidentately bridges the M and H metal sites would be expected to impact on the formation or stability of the tri‐iron centre. Thus, the Y/F subfamily is more likely to contain a binuclear metal centre in contrast to the E/D‐T‐G subgroup which are predicted to have a trinuclear metal centre like *Pm*GH. The boundary between these two subfamilies is not entirely clear‐cut however. For example, RpfG from *X. campestris*, despite phylogenetically clustering within the E/D‐T‐G subgroup, aligns a glycine in place of the E/D residue, as well as a phenylalanine replacing the H‐site metal ligand H189 of *Pm*GH (Fig. 1D). The loss of two out of the eight metal ligands utilized by *Pm*GH to bind the trinuclear Fe centre suggests that RpfG is more likely to possess a binuclear metal ion centre. This phylogenetic analysis, while suggesting substantial diversity within the HD‐GYP family of signalling proteins, at the same time identifies a potential quasi‐equal distribution of putative bi‐ and trinuclear metal centres. Additional crystal structures of representative HD‐GYP domain proteins will be required to clearly understand the structural evolution of this family of PDEs and the requirement for a bi‐ or trinuclear metal centre for catalysis.

### Structural insights into the phosphodiesterase activity of the HD‐GYP domain

The structure of the *Pm*GH‐c‐di‐GMP complex predicts that the M‐site Fe(III) directly interacts with a non‐bridging oxygen of one of the scissile phosphate diesters of the c‐di‐GMP substrate to provide a strong Lewis acid catalyst, while the metal‐activated bridging hydroxide ion of the M‐H Fe pair is the likely nucleophile for the hydrolysis of the scissile bond as similarly proposed for other metallo‐phosphatases (Williams *et al*., 1999). However, this structure does not provide a definitive answer to how the O3′ leaving group is protonated. The closest conserved residues to the O3′ are D183, E185 and K225, with the carboxylate group of E185 situated less than 4 Å away; but the principle role of E185 is expected to be in metal binding. Moreover, the E185A mutant is still capable of hydrolysing c‐di‐GMP although significantly less effectively than the native protein while alanine mutation of either D183 or K225 abolishes enzyme activity (Fig. 1A). In the c‐di‐GMP‐*Pm*GH complex, the D183/K225 pair is over 5 Å away from the O3′ of c‐di‐GMP. As the structure of the c‐di‐GMP‐*Pm*GH complex only contains the G‐site Fe (due to EDTA treatment of crystals), binding of the c‐di‐GMP with an intact trinuclear metal centre is expected to influence the mode of binding of the nucleotide which could place D183 appropriately to act as a general acid for protonation of the O3′ leaving group with K225 stabilizing the unprotonated state of D183. Protonation of O3′ by bound water has been proposed for EAL PDEs as well as for the HD domain phosphohydrolase YfbR (Zimmerman *et al*., 2008; Barends *et al*., 2009; Tchigvintsev *et al*., 2010) but in the structures presented here no suitably positioned water is observed bound.

The complex of c‐di‐GMP with *Pm*GH shows the nucleotide to bind with the guanine bases in a *cis* conformation which differs to EAL domain proteins, in which c‐di‐GMP adopts a more extended conformation (Navarro *et al*., 2009). Similar *cis* conformations for c‐di‐GMP have been observed in other structures, for example in complexes with riboswitches, the degenerate GGDEF domain protein PelD, the c‐di‐GMP effector protein domain PilZ (Habazettl *et al*., 2011; Smith *et al*., 2011; Li *et al*., 2012), and with the human STING proteins (Shu *et al*., 2012), which are stimulators of interferon genes. The *cis*‐conformation together with the space provided by the nucleotide binding cavity is proposed to facilitate the sequential hydrolysis of c‐di‐GMP by HD‐GYP PDEs. Based on the structure of the complex of *Pm*GH with the final reaction product GMP, which shows the 5′‐phosphate of GMP bound to the M‐H Fe pair, c‐di‐GMP hydrolysis may be catalysed by these two sites alone, with the G‐site Fe contributing to the hydrolysis rate. This would invoke primarily a two‐metal‐ion mechanism as seen for other PDEs (Barends *et al*., 2009; Tchigvintsev *et al*., 2010). In this case, the two scissile c‐di‐GMP phosphates would be sequentially hydrolysed by the M‐H Fe pair with the phosphate group which initially binds to the hydroxyl group of Y285 being brought into a similar conformation to the phosphate group bound to the M‐site Fe(III). After hydrolysis of this M‐site bound phosphate group, the hydrolysable phosphate group of 5′‐phosphoguanylyl‐(3′‐5′)‐guanosine (5′‐pGpG), the product of this first hydrolysis step, can be positioned in a similar conformation to the previously hydrolysed phosphate by a rotation around the original twofold axis of the intact c‐di‐GMP. This would then enable the conversion of the now bound 5′‐pGpG to GMP. Equally, it is conceivable that the G‐M Fe pair could also contribute to hydrolysis but the capture of the complex between *Pm*GH and c‐di‐GMP due to the presence only of the G‐site Fe suggests this Fe may play more of a structural role, although clearly a critical one as single alanine mutations of the conserved protein metal ligands of the G‐site Fe renders the protein inactive. In closing, a direct role in catalysis for all three metal sites in *Pm*GH cannot be ruled out as the enzyme has adapted a novel tri‐iron centre with the metal ions closely spaced (≤ 3.64 Å) and single point mutations of any of the eight protein metal ligands abolished activity in all cases except for E185 and D305.The impact of these mutations which would disrupt binding of the M‐H ion pair maybe artificially mitigated by binding of phosphate or carboxylate ions from the cell as the ligands are positioned on the solvent facing side of the metal centre. The mutations do impair the activity of *Pm*GH and thus the predicted distribution of bi‐ and trinuclear centres in the HD‐GYP family may contribute to modulation of PDE activity. Further structural work will be required to decipher unequivocally the roles of the three metal Fe sites which will be aided by a crystal structure of an active binuclear HD‐GYP domain together with a global kinetic analysis of both the bi‐ and tri‐nuclear metal containing HD‐GYP subfamilies identified here.

### Structural insights into the multifunctional roles of HD‐GYP domains

Recent studies have described a regulatory role for protein‐protein interactions involving the HD‐GYP domain protein RpfG. Interaction of RpfG with specific GGDEF domain proteins serves to control motility in *X. campestris* (Ryan *et al*., 2012). Mutational analysis showed that the GYP motif is critical for HD‐GYP::GGDEF complex formation but not necessary for the PDE activity of RpfG against c‐di‐GMP (Ryan *et al*., 2010). The structure of the *Pm*GH HD‐GYP reveals that although the GYP containing loop is surface exposed and well ordered, the Y285 of the GYP motif is placed inside the substrate‐binding pocket, where it H‐bonds to c‐di‐GMP. Therefore, the structural data presented in this study suggests that if GGDEF domains interact directly with Y285, they need to intercalate with the inner side of the HD‐GYP nucleotide‐binding pocket. Such a mechanism would clearly prevent c‐di‐GMP binding and PDE activity, although this has not been observed *in vitro* (Ryan *et al*., 2012). An intriguing alternative is that RpfG acts as a trigger enzyme for protein complex formation and regulation in a similar fashion to the EAL domain protein YciR of *Escherichia coli* (Lindenberg *et al*., 2013). In this scenario, RpfG involvement in protein‐protein complexes would be determined not only by c‐di‐GMP binding but also by conformational alterations associated with c‐di‐GMP degradation, which would be ‘reported’ via the GYP loop.

With the work described here we now have structures of representatives of all domains involved in c‐di‐GMP metabolism. However, further structural work is required to gain a fuller understanding of the relative contributions of the amino acids making up the catalytic site of the enzyme and to understand how the HD‐GYP domain can utilize both bi‐ and trinuclear metal centres to hydrolyse c‐di‐GMP which we propose exist here. Furthermore, the elucidation of the structures of additional HD‐GYP domains from the different classes that we have defined here and of the HD‐GYP domain in complex with the GGDEF domain will be necessary to provide a fuller understanding of the regulatory action of this diverse family of signalling proteins.

## Experimental procedures

### Cloning and protein production

Initial construct design was aided by bioinformatic tools implemented using the OPAL and OPTIC resources as described by Albeck *et al*. (2006) as well as the XTALPRED and HHPRED servers (Söding *et al*., 2005; Slabinski *et al*., 2007). The gene encoding for the GAF/HD‐GYP protein from *Persephonella marina* EX‐H1 (perma_0986; referred to here as *Pm*GH) was optimized for structural studies through mutations of its two cysteines to alanines and deletion of the terminal three amino acids which were predicted to be unstructured (Remmert *et al*., 2012). The construct was synthesized by Genscript inserted in pUC57 and subcloned into pET47b using the STRU‐cloning protocol (Bellini *et al*., 2011) and transformed into *E. coli* BL21 (DE3). BL21 (DE3) cells were grown in LB media and induced with 0.25 mM IPTG; protein overexpression was carried out at 37°C for 1 h. Purification was achieved by Ni^2+^ affinity chromatography using the N‐terminal His6 tag followed by tag cleavage using recombinant HRV 3C protease. Protein was concentrated to 10 mg ml^−1^ for crystallization experiments.

### Crystallization, data collection and structure solution

Diffraction quality crystals were obtained using the hanging drop vapour diffusion method from mixing equal volumes of protein with a precipitant solution made up of 0.1 M MES pH 6.5, 0.9 M succinic acid and 2% PEG 2000. *Pm*GH crystallized in space group I222 with one dimer in the asymmetric unit. Crystals were cryoprotected with ethylene glycol [25% (v/v)] prior to flash‐cooling in liquid nitrogen. The structure was solved using SAD phashing by exploiting the anomalous scattering from the bound iron atoms that were identified by an X‐ray fluorescence spectrum prior to the diffraction experiment (Walsh *et al*., 1999). Native and SAD data at the Fe‐Kα absorption edge were collected on Diamond Light Source beamlines I02 and I24 respectively. Nucleotide‐*Pm*GH complexes were obtained by soaking *Pm*GH crystals soaked overnight at 277 K with 25 mM c‐di‐GMP (BioLog/KeraFast) and 30 mM GMP both in the presence and absence of 100 mM EDTA and data evaluated and collected on Diamond beamlines I02, I03, I04 and I04‐1. The structure of *Pm*GH was determined from a single wavelength anomalous diffraction experiment at the Fe absorption edge determined experimentally on beamline I24 and data extended against a high resolution data set (2.03 Å) collected from a different crystal using beamline I02. All data were processed using XDS within XIA2 (Kabsch, 2010; Winter, 2010) and the Fe substructure was determined using SHELX (Sheldrick, 2010). Building of the structure was aided by automated procedures in Buccaneer (Cowtan, 2006) and manual building was performed with COOT (Emsley *et al*., 2010). The structure was refined with REFMAC5 (Murshudov *et al*., 1997) and validated with MolProbity (Chen *et al*., 2010). Figures of structures were prepared with PyMOL (Schrodinger, 2010).

### Alteration of residues in the *Pm*GH HD‐GYP domain by mutagenic PCR

Site‐directed mutagenesis to introduce the alterations in residues involved in metal binding (E185A, H189A, H221A, D222A, H250A, H276A, H277A, D305A and D305A), substrate binding (Y285A, R314A and K317A) and other residues of interest (D183A, K225A, G284A, P286A, I294A and D308A) was done by using mutagenic PCR in a two‐step protocol as previously described (Ryan *et al*., 2006; 2010). In the first round of PCR, two separate reactions were carried out by using the forward and reverse primers together with one of a pair of primers of complementary sequence carrying the desired alteration and the HD‐GYP construct in pET47b as template. (Mutagenic primer sequences will be given upon request.) The products of the first round of PCR were used as templates for a second round of PCR with forward and reverse primers.

### Enzymatic assays on the HD‐GYP domain and its variants

The assay buffer and reaction conditions were as described elsewhere (Ryan *et al*., 2006). Briefly, a standard reaction mixture contained 20 μg of protein, 50 mM Tris·HCl (pH 7.6), 10 mM MgCl_2_, 10 mM MnCl_2_, 0.5 mM EDTA and 50 mM NaCl in a total volume of 600 μl. The assay mixture was warmed to 37°C before the reaction was started by the addition of 27 μl of substrate to give a final concentration of 100 μM. Aliquots of 200 μl were withdrawn to a sterile Eppendorf tube at the indicated time points, and the assay was terminated by placing the tube in a boiling water bath for 3 min. After centrifugation at 15 000 *g* for 2 min, the supernatant was filtered through a 0.22 μm filter before analysis by reverse‐phase HPLC on a Hewlett–Packard Model 1090 Series II HPLC system. Samples of 50 μl were injected into a SunFire C‐18‐T column (150 × 4.6 mm; Waters) and fractionated by using 2% (v/v) acetonitrile/98% Na phosphate buffer (pH 5.8) under isocratic condition at a flow rate of 0.7 ml min^−1^. Nucleotides were detected at a wavelength of 252 nm.

### Accession numbers

The co‐ordinates and structure factors have been deposited in the Protein Data Bank, http://www.pdb.org PDB ID codes 4MCW (*Pm*GH), 4MDZ (complex with c‐di‐GMP) and 4ME4 (complex with GMP).

## Supplementary Material

Supporting Information
