# Supplementary material for: Crystal structure of an HD‐GYP domain cyclic‐di‐GMP phosphodiesterase reveals an enzyme with a novel trinuclear catalytic iron centre
Source: Mol Microbiol. 2013 Nov 24;91(1):26–38. doi: 10.1111/mmi.12447 (PMC4159591; doi:10.1111/mmi.12447)
Supplement: Supplementary file 1 — Supporting Information [file mmi-91-26-s1.pdf]

Supporting Information

Bellini et al.

Crystal structure of an HD-GYP domain cyclic-di-GMP phosphodiesterase reveals an enzyme with a novel trinuclear catalytic iron center

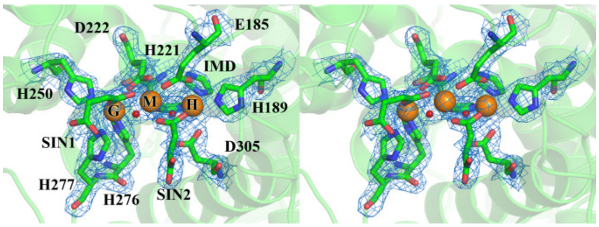

Figure S1. Stereoview of the triiron active site of *Pm*GH. Metal ligands are shown in ball and stick, coloured by atom type with carbon in green, nitrogen blue and oxygen red. The crystallization buffer contributes three metal ligands via two succinates and an imidizole ion which are labelled SIN1, SIN2 and IMD, respectively. The tri-iron metal centre is represented as for Fig. 1 with the metal μ-hydroxo bridges shown as red spheres. Electron density is from a 2m*F*oD*F*c map contoured at the 3 σ level, except for SIN1-2 which are contoured at the 2 σ level for clarity.

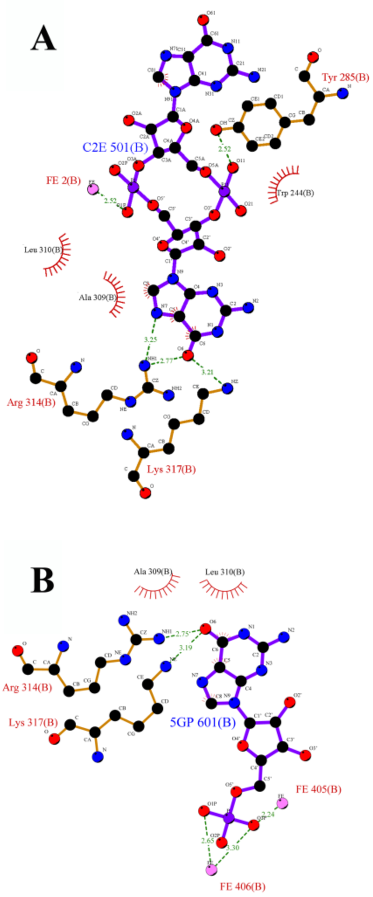

Figure S2. Ligplot diagrams. Hydrogen bonding, non-bonding and metal interactions for (A) c-di-GMP and (B) GMP bound to *Pm*GH.

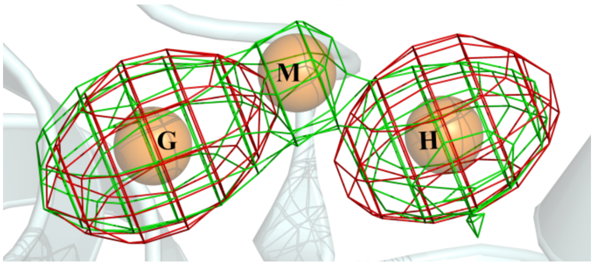

Figure S3. Anomalous difference maps for the trinuclear metal centre (G, M and H sites) of *Pm*GH expressed in minimal media supplemented only with $MnCl_2$. Green: Iron specific anomalous difference map . Red: Manganese anomalous difference map. Both maps are contoured at 0.043 eÅ^{-3}. These data show the presence of both Mn and Fe at the G and H metal sites but no Mn anomalous signal for the M site which indicates it is specific for Fe.
